# Serum sickness-like reaction to D-mannose supplement: a case report

**DOI:** 10.1186/s12887-024-04753-8

**Published:** 2024-06-22

**Authors:** Emma R. Plante, Charles Ekwunwa, Michelle C. Maciag, Diego Illanes

**Affiliations:** 1 Department of Gynecology and Urogynecology, Milford Regional Medical Center, 14 Prospect Street, Milford, MA 01757 USA; 2 Department of Obstetrics and Gynecology, Tufts University School of Medicine, 800 Washington St, Boston, MA 02111 USA; 3 Asthma and Allergy Affiliates, 114R Highland Ave, Salem, MA 01970 USA; 4 Division of Allergy and Immunology, Boston Children’s Hospital, 300 Longwood Ave, Boston, MA 02115 USA

**Keywords:** Serum sickness-like reaction, SSLR, Serum sickness, D-mannose, UQora

## Abstract

**Background:**

Serum Sickness-Like Reaction (SSLR) is an immune response characterized by rash, polyarthralgias, inflammation, and fever. Serum sickness-like reaction is commonly attributed to antibiotics, anticonvulsants, and anti-inflammatory agents.

**Case presentation:**

A 16-year-old female with a history of overactive bladder and anemia presented with a diffuse urticarial rash, headaches, joint pain, and swelling for three days. Her medications included oral contraceptive pills, iron, mirabegron, UQora, and a probiotic. Physical examination revealed a diffuse urticarial rash, and her musculoskeletal exam revealed swelling and tenderness in her wrists. She was evaluated by her pediatrician and started on a 7-day course of prednisone, as well as antihistamines. Her CBC, basic metabolic panel, liver function panel, Lyme titers, and urinalysis were all within normal limits. With concern for hypersensitivity reaction to medication, all medications were discontinued. Nine days after symptom onset, the patient was evaluated by an allergist, who confirmed her presentation was consistent with serum sickness-like reaction. Her symptoms resolved, and her medications were re-introduced sequentially over several months. Restarting UQora, however, triggered a recurrence of her symptoms, and it was identified as the culprit medication. Consequently, UQora was permanently discontinued, and the patient has remained symptom-free.

**Conclusions:**

This case report describes the first documented case of serum sickness-like reaction caused by UQora (active ingredient D-mannose). D-mannose is a monosaccharide, and it is frequently promoted to prevent urinary tract infections. While the clinical features and timeline in this case were typical of serum sickness-like reaction, UQora as the trigger was highly unusual. Clinicians should be aware of the diverse triggers of serum sickness-like reaction and the importance of prompt identification and management to enhance patient safety. Further research is necessary to better understand the potential therapeutic applications of D-mannose, as well as the potential risks and interactions.

## Background

Serum sickness and serum sickness-like reaction (SSLR) are both immunological conditions characterized by rash, polyarthralgia, and fever. Serum sickness is a type III hypersensitivity reaction where immune complexes develop between human and non-human proteins (such as vaccines, immunomodulators, and anti-venoms) [1–3]. In contrast, SSLR has a similar clinical presentation but usually occurs in response to a new medication that triggers the development of drug-specific immune complexes [4, 5]. Antibiotics are a common cause of SSLR (particularly the second-generation cephalosporin cefaclor), as are anticonvulsants, and anti-inflammatory agents [6]. The underlying immune mechanisms involved in SSLRs are less well-understood but believed to differ from those leading to serum sickness. Here we present a case of a 16-year-old female patient who developed a SSLR in response to the D-mannose-containing over-the-counter supplement, Uqora, used to promote bladder health.

## Case presentation

A 16 year-old white female with a history of overactive bladder and iron-deficiency anemia presented to her pediatrician with three days of diffuse urticarial rash, headaches, joint tenderness, and synovitic swelling. The rash started on her arms, extended to her torso, and ultimately spread to her entire body. She denied fever, cough, sore throat, oropharyngeal swelling, or shortness of breath. There was no clinical evidence of viral respiratory illness. Her diet, detergents, soaps, and skin care regimen had no recent changes. She had no prior history of urticarial reactions. Approximately three weeks prior to presentation, she began using the over-the-counter bladder supplement Uqora (active ingredient D-mannose) twice daily. Her other medications (all of which she had been taking for many months) included combined oral contraceptive pills (drospirenone 3 mg / ethinyl estradiol tablets 0.02 mg), mirabegron 25 mg daily, Slow Fe 20 mg daily, and a probiotic.

Physical examination demonstrated normal vital signs, normal ear, nose, and throat exam, normal cardiorespiratory exam, soft, non-tender abdomen, no hepatosplenomegaly, no lymphadenopathy. Skin examination showed a diffuse, urticarial rash. A musculoskeletal exam revealed mild swelling and tenderness in bilateral wrists with no overlying erythema. She also exhibited joint tenderness without swelling in bilateral ankles. Laboratory tests were performed by her pediatrician and were normal, including a complete blood count, comprehensive metabolic panel, Lyme titers, and urinalysis. (Tables 1, 2, 3 and 4)

**Table 1:** Comprehensive metabolic panel

Test	Results	Normal Range
Sodium	139 mmol/L	136–145 mmol/L
Potassium	4.6 mmol/L	3.5–5.2 mmol/L
Chloride	104 mmol/L	95–106 mmol/L
Carbon Dioxide, Total	24 mmol/L	20–31 mmol/L
Urea Nitrogen	11 mg/dL	9–23 mg/dL
Creatinine	0.68 mg/dL	0.5–1.30 mg/dL
Glucose	91 mg/dL	74–106 mg/dL
Albumin	4.2 g/dL	3.5–4.8 g/dL
Total Protein	6.8 g/dL	5.7–8.2 g/dL
Calcium	8.9 mg/dL	8.7–10.4 mg/dL
Alkaline Phosphatase	86 U/L	36–210 U/L
Bilirubin, Total	< 0.2 mg/dL	0.0–1.0 mg/dL
AST (SGOT)	22 U/L	6–40 U/L
ALT (SGPT)	17 U/L	10–49 U/L
Globulin	2.6 g/dL	1.9–4.1 g/dL
eGFR	Not calculated (under 18)	> 60 mL/min/1.73m2
Anion Gap	11 mmol/L	3–17 mmol/L

**Table 2:** CBC (W/DIFF AND PLT)

Test	Results	Normal Range
White Blood Cells	9.57 K/uL	4.5–13.5 K/uL
RBC	4.65 M/uL	4.1–5.1 M/uL
Hemoglobin	13.0 g/dL	12.0–16.0 g/dL
Hematocrit	38.9%	36–46%
PLT	259 K/uL	135–400 K/uL
MCV	83.7 fL	78–102 fL
MCH	28.0 pg	27–34 pg
MCHC	33.4 g/dL	31.5–36.5 g/dL
RDW by Automated Count	12.7%	11.9–14.8%
Platelet Mean volume	11.2 fl.	9.6–12 fl.
Nucleated RBC	0.00 WBCs	0/100 WBCs
Diff Method	Auto	
Neutrophils %	84.1%	
Lymphs	13.2%	
Monos	2.3%	
Eosinophil Relative	0.0%	
Basosphils	0.0%	
% Immature Granulocyte	0.4%	
Absolute Neutrophils (Auto)	8.05 K/uL	1.8–8 K/uL
Lymph #	1.26 K/uL	1.5–6.5 K/uL
Mono #	0.22 K/uL	0.1–0.9 K/uL
EOS	0.00 K/uL	0–0.4 K/uL
Baso #	0.00 K/uL	0–0.2 K/uL
Granulocytes	0.04 K/uL	0–0.03 K/uL

**Table 3:** Lyme disease AB W/REFL

Test	Results	Normal Value
Lyme AB IGG	Negative	Negative
Lyme IgM	Negative	Negative

**Table 4:** Urinalysis auto only

Test	Results	Normal Range
Color, Urine	Yellow	
Clarity, Urine	Cloudy	
Glucose (MG/DL) in Urine	Negative	Negative
Ketones, Urine	Negative	Negative
Specific Gravity, Urine	1.030	1.001–1.035
Blood, Urine, POC	Moderate	Negative
pH, Urine	6.0	4.6-8.0
Protein, Urine	Trace	Negative
Nitrite, Urine	Negative	Negative
Leukocytes, Urine	Negative	Negative

On the basis of history, physical exam, and laboratory data, a clinical diagnosis of SSLR was made. All of her medications were discontinued, and she was treated with a course of oral prednisone and cetirizine. She was also given an EpiPen and educated on its use. Her symptoms improved significantly within a few days. Initially, the exact trigger for her SSLR was unclear. Once her symptoms resolved, she was instructed to restart her medications one at a time. Over a period of many months, she reintroduced each medication two weeks apart. She remained asymptomatic until she resumed the Uqora, which triggered the recurrence of her urticarial rash and joint swelling that night and into the following morning. At that point, it became clear that the bladder health supplement was the precipitating agent for her SSLR, and it was permanently discontinued. Her symptoms resolved completely and have not returned despite continuing all of her other previous medications.

## Discussion and conclusions

Both serum sickness and SSLR are immunologically mediated through the inflammatory cascade, although the specific immune mechanisms associated with SSLR are less well-characterized [1–3, 5]. Serum sickness and SSLR have similar clinical presentations: urticarial rash, joint pain and swelling, and fevers are the hallmark features. Cases of serum sickness tend to be more severe and may result in end organ dysfunction such as renal failure, vasculitis, uveitis, and neuropathy. Serum sickness occurs more often in adults, and vaccines and immunomodulators are the most common culprits [7, 8]. 

In contrast, SSLR occurs more often in children, and is usually triggered by medications (most frequently antibiotics, anticonvulsants, and anti-inflammatory agents) [9, 10]. SSLR is a clinical diagnosis, and the development of symptoms usually begins approximately 1–4 weeks after exposure to the offending agent. Re-exposure to the offending agent in a SSLR will often trigger more immediate symptoms, as seen in our patient. Furthermore, in many cases, subsequent exposure results in a more severe presentation [1]. Most cases of SSLR are self-limited and resolve once exposure to the antigenic substance is discontinued.

The most critical component of the treatment regimen for serum sickness and SSLR is discontinuing the offending agent. Withdrawing the medication alone may be sufficient to resolve the condition. Nevertheless, glucocorticoids are frequently used to treat both diseases. They appear to be most beneficial for patients with more severe symptoms, particularly those with evidence of end-organ damage. The evidence for the use of glucocorticoids, however, comes from case reports and small observational studies [1]. Oral regimens are typically sufficient. Rarely, in the most severe cases, patients are treated with intravenous methylprednisolone. Our patient did not develop severe sequelae and was successfully treated with an outpatient course of oral steroids.

In this case, the clinical features and timeline were classic for SSLR. The trigger, however, the bladder supplement Uqora, was very unusual and has not been previously described as a cause for this condition. In reviewing our patient’s medications, mirabegron (brand name Myrbetriq), a β3-adrenergic receptor agonist, has been linked to a single case of SSLR [11]. We ruled out mirabegron as our patient’s SSLR trigger, however, because she resumed this medication without any adverse reaction. Similarly, intravenous administration of iron-dextran has been associated with a single reported case of serum sickness [12]. We excluded iron as the cause of SSLR because our patient was taking iron orally and resumed her iron supplement without incident.

Ultimately, it became apparent that the bladder supplement, whose active ingredient is D-mannose, was responsible for our patient’s SSLR because her urticarial rash reappeared within one day of resuming it. It is possible that D-mannose was not the ingredient that caused the reaction, but it was the most likely culprit as it is the active ingredient in this product. Additionally, it is possible that an active ingredient in Uqora could have interacted with another one of this patient’s medications. A third possibility is that she had a prolonged viral response. This explanation is much less likely because after discontinuing the bladder supplement, her symptoms resolved, and upon resumption of the supplement, her symptoms returned.

D-mannose is a monosaccharide that naturally exists in plants and fruits. Its concentration in human blood is approximately 1/100th that of glucose [13]. Despite limited studies, D-mannose is often promoted as a natural method to prevent urinary tract infections by blocking bacterial adherence to uroepithelial cells [14]. Additionally, emerging data suggests that D-mannose may have immunoregulatory functions and is being explored as a possible treatment adjunct for certain malignancies, autoimmune conditions, and other inflammatory disorders [13, 15–17]. 

To our knowledge, this case is the first reported case of a SSLR associated with a D-mannose-containing product. Transparent labeling for dietary supplements and the provision of safety information are essential to promote informed decision-making among consumers seeking relief from common bladder complaints. Further research is needed to better understand the potential therapeutic applications of D-mannose, as well as the potential risks and interactions.

## Data Availability

All data generated or analysed during this study are included in this published article.

## Abbreviations

SSLR: serum sickness-like reaction
