# Correction: Serum sickness-like reaction to D-Supplement: A Case Report

**DOI:** 10.1186/s12887-024-04962-1

**Published:** 2024-07-26

**Authors:** Emma R. Plante, Charles Ekwunwa, Michelle C. Maciag, Diego Illanes

**Affiliations:** 1 Department of Gynecology and Urogynecology, Milford Regional Medical Center, 14 Prospect Street, Milford, MA 01757 USA; 2 Department of Obstetrics and Gynecology, Tufts University School of Medicine, 800 Washington St, Boston, MA 02111 USA; 3 Asthma and Allergy Affiliates, 114R Highland Ave, Salem, MA 01970 USA; 4 Division of Allergy and Immunology, Boston Children’s Hospital, 300 Longwood Ave, Boston, MA 02115 USA

**Correction: MC Pediatr24**,** 404 (2024)**

10.1186/s12887-024-04753-8

Following publication of the original article [1], the authors identified an error in the article title. The correct and incorrect article title are given below. The original article has been corrected.

Correct article title:

Serum sickness-like reaction to D-Mannose Supplement: A Case Report.

Incorrect article title:

Serum sickness-like reaction to D-supplement: a case report.

## References

[1] Plante ER, Ekwunwa C, Maciag MC, et al. Serum sickness-like reaction to D-supplement: a case report. BMC Pediatr. 2024;24:404. 10.1186/s12887-024-04753-8. 38909179 10.1186/s12887-024-04753-8 PMC11193288

